# Altered Balance of Pro-Inflammatory Immune Cells to T Regulatory Cells Differentiates Symptomatic From Asymptomatic Individuals With Anti-Nuclear Antibodies

**DOI:** 10.3389/fimmu.2022.886442

**Published:** 2022-06-30

**Authors:** Rashi Gupta, Emma Vanlieshout, Kieran Manion, Dennisse Bonilla, Michael Kim, Carolina Muñoz-Grajales, Carol Nassar, Sindhu R. Johnson, Linda T. Hiraki, Zareen Ahmad, Zahi Touma, Arthur Bookman, Joan E. Wither

**Affiliations:** ^1^ Department of Immunology, University of Toronto, Toronto, ON, Canada; ^2^ Schroeder Arthritis Institute, Krembil Research Institute, University Health Network, Toronto, ON, Canada; ^3^ Department of Medicine, University Health Network, University of Toronto, Toronto, ON, Canada; ^4^ Toronto Scleroderma Program, Department of Medicine, Toronto Western and Mount Sinai Hospitals, University of Toronto, Toronto, ON, Canada; ^5^ The Hospital for Sick Children, Department of Paediatrics, University of Toronto, Toronto, ON, Canada; ^6^ University of Toronto Lupus Clinic, Centre for Prognosis Studies in Rheumatic Diseases, Schroeder Arthritis Institute, University Health Network, Toronto, ON, Canada; ^7^ Division of Rheumatology, Schroeder Arthritis Institute, University Health Network, Toronto, ON, Canada

**Keywords:** b cells, monocytes, t cells, dendritic cells, anti-nuclear antibodies, systemic autoimmune rheumatic diseases, interferon-alpha, t regulatory cells

## Abstract

Systemic Autoimmune Rheumatic Diseases (SARDs) are characterized by the production of anti-nuclear antibodies (ANAs). ANAs are also seen in healthy individuals and can be detected years before disease onset in SARD. Both the immunological changes that promote development of clinical symptoms in SARD and those that prevent autoimmunity in asymptomatic ANA^+^ individuals (ANA^+^ NS) remain largely unexplored. To address this question, we used flow cytometry to examine peripheral blood immune populations in ANA^+^ individuals, with and without SARD, including 20 individuals who subsequently demonstrated symptom progression. Several immune populations were expanded in ANA^+^ individuals with and without SARD, as compared with ANA^-^ healthy controls, particularly follicular and peripheral T helper, and antibody-producing B cell subsets. In ANA^+^ NS individuals, there were significant increases in T regulatory subsets and TGF-ß1 that normalized in SARD patients, whereas in SARD patients there were increases in Th2 and Th17 helper cell levels as compared with ANA^+^ NS individuals, resulting in a shift in the balance between inflammatory and regulatory T cell subsets. Patients with SARD also had increases in the proportion of pro-inflammatory innate immune cell populations, such as CD14^+^ myeloid dendritic cells, and intermediate and non-classical monocytes, as compared to ANA^+^ NS individuals. When comparing ANA^+^ individuals without SARD who progressed clinically over the subsequent 2 years with those who did not, we found that progressors had significantly increased T and B cell activation, as well as increased levels of LAG3^+^ T regulatory cells and TGF-ß1. Collectively, our findings suggest that active immunoregulation prevents clinical autoimmunity in ANA^+^ NS and that this becomes impaired in patients who progress to SARD, resulting in an imbalance favoring inflammation.

## Introduction

The anti-nuclear antibody (ANA)-associated Systemic Autoimmune Rheumatic Diseases (SARD), which include Systemic Lupus Erythematosus (SLE), Sjögren’s Syndrome (SS), and Systemic Sclerosis (SSc), are chronic multi-system autoimmune diseases with a significant morbidity and mortality. Although each of these conditions has some distinctive autoantibodies (autoAbs) and clinical features, there is considerable overlap in the types of autoAbs produced and clinical symptoms, suggesting a shared etiology. This is supported by studies showing numerous shared genetic risk factors (1–5) and a high prevalence of elevated levels of interferon (IFN)-induced gene expression (6–12).

Since SARD can often present with life-threatening inflammation and/or irreversible damage, there is tremendous interest in defining at-risk individuals and initiating therapy early to prevent these poor outcomes. To achieve this, it is necessary to have a highly accurate biomarker for impending disease and knowledge of the key immune events to target. A characteristic feature of SARD is a prolonged preclinical phase in which ANAs can be seen in the absence of clinical symptoms (13–16). While this observation suggests that ANAs could be used to identify at-risk individuals, ANAs, as detected by immunofluorescence using HEp-2 as a substrate, are seen in ~20% of healthy women (12), only a small subset of whom (estimated at 5-8%) will transition to SARD. Thus, additional biomarkers are required to identify ANA positive (ANA^+^) individuals at high risk of impending progression. In addition, little is known about the immunologic features that differentiate asymptomatic ANA^+^ individuals from those with SARD, and progressors from non-progressors.

To address these knowledge gaps, our laboratory has been recruiting and longitudinally following a unique cohort of ANA^+^ individuals lacking a SARD diagnosis. In a previous study, we characterized several B and T cell phenotypes in the peripheral blood of these subjects, contrasting them with those seen in ANA^-^ healthy controls and early SARD patients (17). This led to the surprising observation that ANA^+^ individuals lacking a SARD diagnosis had increased proportions of activated B and T cells, similar to that observed in early SARD. Indeed, in that original study, except for a trend to increased activation in ANA^+^ individuals with SARD as compared to those without, no distinctive immunologic differences were seen between these two groups. In this study, we examined a broader array of immune populations in an effort to define the key immunologic differences that discriminate between ANA^+^ individuals with and without a SARD diagnosis, and to characterize the immunologic changes that distinguish ANA^+^ individuals who demonstrate subsequent clinical progression from those who do not.

## Materials and Methods

### Subjects and Data Collection

ANA^+^ individuals (≥1:160 or 1:80 with a specific autoAb) were recruited from the Toronto Western and Mount Sinai Hospital Rheumatology Clinics, where they had been referred for evaluation because of a positive ANA test. Following assessment by one of the participating rheumatologists, patients were stratified into three groups based upon the presence of SARD clinical diagnostic criteria [1997 American College of Rheumatology (ACR) criteria for SLE (18), 2013 ACR/European League Against Rheumatism (EULAR) criteria for SSc (19), or the revised 2016 ACR/EULAR criteria for SS (20)], as follows: (1) asymptomatic ANA^+^ (ANA^+^ NS), with no clinical SARD criteria; (2) undifferentiated connective tissue disease (UCTD), with at least one clinical symptom of SARD but who did not meet criteria for SARD diagnosis; or (3) early SARD. All SARD patients included within the study met disease classification criteria, were within the first 2 years of diagnosis, and were not taking corticosteroids or disease-modifying anti-rheumatic drugs, with the exception of hydroxychloroquine. For patients seen after 2015, yearly follow-up was offered to monitor any potential disease progression, and all patients with at least 2 years of follow-up care were included in the study, contrasting progressors and non-progressors. Clinical progressors were defined based upon development of new clinical SARD criteria or new organ involvement characteristic for SARD, within 2 years of initial assessment. Sex-matched ANA^-^ healthy controls (ANA^-^ HC) were recruited from hospital and laboratory personnel. Patients provided information on a family history of rheumatic disease using a validated questionnaire (21). The study was approved by the Research Ethics Boards of the two hospitals and all participants signed informed consent.

### Cellular Characterization

Peripheral blood mononuclear cells (PBMCs) were isolated from whole blood collected in sodium-heparin tubes over a Ficoll/Hypaque (GE Healthcare) gradient, treated to remove residual red blood cells, and immediately stained, or archived in Liquid N_2_ (in CryoStor^®^) and subsequently stained immediately following thawing. Prior to staining with various combinations of directly-conjugated monoclonal Abs, the cells (5 x 10^5^/stain) were incubated with viability dye (Fixable Far-Green Dead Cell Stain, Invitrogen) for 30 minutes on ice. The Abs used for staining were as follows: mouse anti-human, TBET-PE (4B10), FOXP3-PE (206D), CD56-PE (5.1H11), CD4-PerCP (SK3), IgD-PerCP (IA6-2), CD123-PerCPCy5.5 (6H6), CD11c-PeCy7 (3.9), CD38-PeCy7 (HB-7), CD21-APC (Bu32), CXCR3-APC (G025H7), HELIOS-APC (22F6), CD16-APC (B73.1), CD27-APC/Fire750 (M-T271), CD3-APC/Fire750 (SK7), CD19-BV421 (H1B19), PD1-BV421 (EH12.2H7), CD138-BV605 (MI15), CD20-BV605 (2H7), CXCR5-BV605 (J252D4), CD25-BV605 (2A3), and CD86-BV605 (BU63) from Biolegend; and mouse anti-human CCR6-PE (11A9), CD3-PeCy7 (SK7), CD19-APC-H7 (SJ25C1), CD45RA-APC/Fire750 (HI100), CD20-APC-H7 (2H7), LAG3-BV421 (T47-530), CD14-BV421 (MøP9), and HLADR-BV605 (646-6) from BD Biosciences. Staining for intracellular FOXP3 and HELIOS was performed using the Human FOXP3 Buffer Set (BD Biosciences) for fixation and permeabilization, according to the manufacturer’s protocol. Events were acquired using a three-laser LSRII or FACSCanto (BD Biosciences) flow cytometer, with fluorescence-minus-one (FMO) controls being used as negative staining controls. The data was analyzed using FlowJo software (BD Biosciences).

### Cytokine Measurements

For measurement of transforming growth factor beta-1 (TGF-β1), freshly thawed heparinized plasma (stored at -80°C and not previously thawed) was activated by adding 5 µL of 1.0 M HCl to 10 µL of plasma, and incubated for 10 minutes at room temperature. The reaction was then neutralized by addition of 5 µL of 1.2 M NaCl/0.5M HEPES and the resultant mixture was diluted to a final volume of 400 µL with diluent reagent. The concentration of TGF-ß1 in the diluted activated plasma (100 µL per well, in duplicate) was measured using a human TGF- ß1 DuoSet ELISA Kit and Ancillary Reagent Kit 1 (R&D Systems), with the optical density being read at 450 nm using a FLUOStar^®^ Omega microplate reader (BMG Labtech). IFN5 scores were determined by measuring the expression levels of five IFN-induced genes (*EPSTI1, IFI44L, LY6E, OAS3*, and *RSAD2*) in whole peripheral blood archived in Tempus tubes (Applied Biosystems), using a custom NanoString (NanoString Technologies) (12, 17). Log_2_ normalized expression levels of the 5 genes were summed to generate a composite IFN5 score. Serum IFN-α was measured using patient serum collected and archived at −80°C at the time of recruitment, as previously described (12).

### Measurement of autoAbs

ANAs were quantified by indirect immunofluorescence using the Kallestad^®^ HEp-2 kit (BioRad), through the University Health Network laboratory. The serum levels of 11 specific autoAbs (anti-dsDNA, -chromatin, -Ro, -La, -Sm, -SmRNP, -RNP, -Jo-1, -Scl-70, -centromere, and -ribosomal P), were quantified using the Bioplex^®^ 2200 ANA Screening System (BioRad), with the company’s suggested cut-offs being used to define a positive test. AutoAb testing was performed on all HCs, and those meeting the entrance criteria were re-classified into the asymptomatic ANA^+^ group. HCs with a positive ANA <1:160 or found to have any specific autoAb in the absence of a positive ANA were excluded from the study. Ro60 and Ro52 Abs were measured using an autoantigen microarray, as previously reported (22).

### Data Analysis

The Kruskal-Wallis test was used for statistical comparisons of differences between three or more groups, followed by Dunn’s post-test for multiple comparisons. Comparisons between two groups were performed using the Mann-Whitney test. The strength of correlation between two variables was assessed using Spearman’s correlation coefficient, with the lines that visually display these associations being computed by linear regression analysis. All statistical analyses were performed using GraphPad Prism Software, Version 8 (San Diego, CA, USA), except for the correlation matrices, which were produced in R using the corrplot (v0.84) package. For statistical tests, asterisks indicate a p value of <0.05 (*), <0.01 (**), <0.001 (***), or <0.0001 (****).

## Results

### The T Helper Cell Phenotype Differs Between ANA^+^ Individuals With and Without a SARD Diagnosis

We have previously shown that ANA^+^ NS and UCTD patients share a number of B cell activation phenotypes and increases in the proportion of T follicular helper cells with early SARD patients (17). However, the functional characteristics of the expanded Tfh population and many innate immune populations were not examined. Therefore, to further explore the immunologic differences between symptomatic and asymptomatic ANA^+^ individuals, the current study was performed. **Supplementary Table 1** outlines the demographic characteristics of the subjects, the majority of whom did not overlap with the previously published study.

Although our ANA^+^ NS subjects lacked clinical SARD criteria, they could have other clinical symptoms not attributable to SARD. The ANA testing for these individuals was performed for the following reasons: non-inflammatory arthritis/arthralgias (40%, mostly osteoarthritis and fibromyalgia), sicca symptoms in the absence of objective signs of dryness (15%), healthy mother with a child with congenital heart block or neonatal lupus (14%), urticaria/non-specific rash (11%), family history of autoimmunity (7%), recruitment to the study as a healthy control (6%), and other (7%). All UCTD patients had a least one clinical symptom of SARD, but lacked sufficient disease classification criteria for a diagnosis of SARD. These symptoms included: Raynaud’s phenomenon (38%), inflammatory arthritis (19%), abnormal nailfold capillaries (17%), objective ocular signs (12%), photosensitivity (10%), objective oral signs (8%), puffy fingers (6%), pericarditis (4%), interstitial lung disease (4%), malar rash (4%), ITP/TTP (4%), alopecia (4%), oral ulcers (2%), chilblains (2%), calcinosis (2%), esophageal dysmotility (2%), calcinosis (2%), and oral ulcers (2%). SARD patients had to meet objective disease classification criteria for diagnosis (see *Materials and Methods*). 

The subjects were predominantly female with similar proportions in all groups. However, ANA^−^ HCs were significantly younger than ANA^+^ NS and UCTD patients. There were no significant differences between groups in the ethnicity of the subjects, with the majority of subjects in each group being Caucasian. In all of the ANA^+^ groups, the majority of subjects had an ANA titer of 1:640 or greater, but SARD patients had a larger number of nuclear antigen autoantibody specificities (as determined by the Bioplex^©^) when compared to the other ANA^+^ groups.

**Figure 1:**
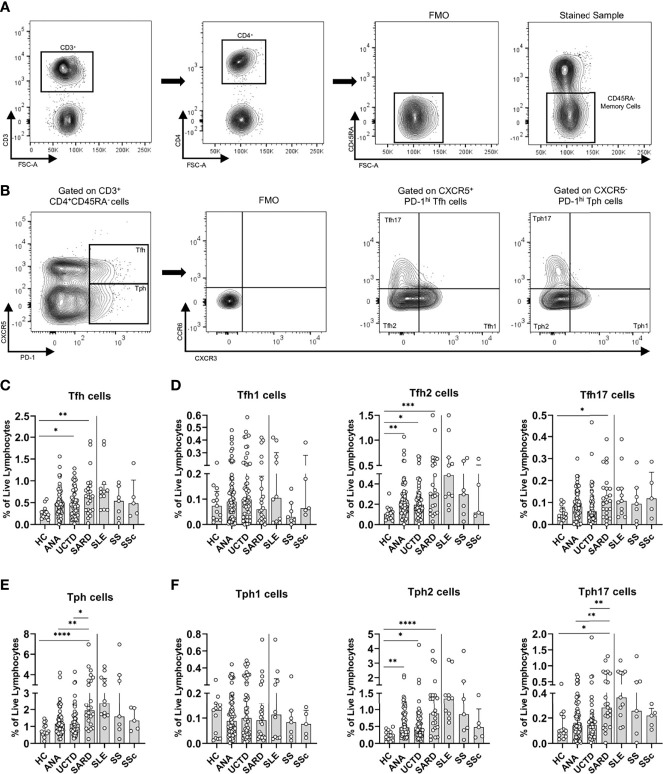
Asymptomatic anti-nuclear antibody positive (ANA^+^) individuals lacking a diagnosis of systemic autoimmune rheumatic diseases (SARD) have abnormalities in T helper subsets that are amplified in symptomatic patients with early SARD. **(A)** Gating strategy for identification of (CD3^+^CD4^+^CD45RA^-^) memory T cells from the peripheral blood mononuclear cells of a representative ANA^+^ patient. **(B)** Gating strategy for identification of T follicular helper (Tfh, PD-1^hi^CXCR5^+^) and T peripheral helper (Tph, PD-1^hi^, CXCR5^-^) cells and the Th1 (CXCR3^+^, CCR6^-^), Th2 (CXCR3^-^, CCR6^-^), and Th17 CXCR3^-^, CCR6^+^) subsets within these populations. **(C, D)** The proportions of Tfh cells and the individual Tfh subsets within the memory T compartment stratified by subject group. **(E, F)** The proportions of Tph cells and the individual Tph subsets within the memory T compartment stratified by subject group. The solid vertical line in each plot separates the groups that were statistically compared to one another from the individual SARD on the right, which were not statistically compared to any group. Bars represent the median with interquartile range. Each data point represents an individual subject. Statistical significance was determined using the Kruskal-Wallis test with Dunn’s *post-hoc* test for multiple comparisons; *p ≤ 0.05, **p ≤ 0.01, ***p ≤ 0.001, ****p ≤ 0.0001. HC, ANA^-^ healthy control; ANA, asymptomatic ANA^+^; UCTD, undifferentiated connective tissue disease; SARD, systemic autoimmune rheumatic disease; SLE, systemic lupus erythematosus; SS, Sjögren’s syndrome; SSc, systemic sclerosis.

Although most studies have shown an increase in Tfh cells in SARD, there has been inconsistency between studies as to which sub-populations of cytokine-producing cells are increased (23–29). To determine whether the cytokine profile of Tfh cells in ANA^+^ NS and UCTD patients is similar to that seen in early SARD, PBMCs were stained to identify Tfh (CD3^+^CD4^+^CD45RA^-^PD1^hi^CXCR5^+^) cells. The proportion of cells with a Th1, Th2 or Th17 phenotype was then determined by staining with anti-CXCR3 and CCR6 monoclonal Abs, with the CXCR3^+^CCR6^-^, CXCR3^-^CCR6^-^, and CXCR3^-^CCR6^+^ populations being enriched for Th1, Th2, and Th17 cells (representative gating shown in **Figures 1A, B**), as previously reported (30).

Compatible with previous reports of increased Tfh cells in SLE, SS, and SSc, there was a significant expansion of Tfh cells in early SARD patients as compared to ANA^-^ HC, and as observed in our previous study, this was also seen to a lesser extent in ANA^+^ NS or UCTD patients (**Figure 1C**). The increases in Tfh cells in early SARD occurred in the Th2 and Th17 subsets, with no difference in the proportion of Th1 cells, as compared to ANA^-^ HC. ANA^+^ NS and UCTD patients also showed a trend to increased proportions of Tfh cells, which was smaller than that seen in SARD, and which appeared to result from small increases in the Th1 and Th17 subsets, together with a significant increase in the Th2 cell subset (**Figure 1D**).

Recently, a novel extra-follicular T helper subset termed T peripheral helper (Tph) cells that shares many properties with Tfh cells but lacks expression of CXCR5 (representative Tph gating shown in **Figure 1B**) was found to be increased in SLE and SS (31–33). This cell subset was increased in early SARD, at significantly higher levels than those seen in ANA^+^ NS and UCTD patients (**Figure 1E**). As was observed for Tfh in early SARD, the increase in Tph cells was attributable to increases in the proportion of the Th2 and Th17 subsets within this population (**Figure 1F**). The proportion of Tph2 cells was also significantly increased in ANA^+^ NS and UCTD patients, but the magnitude of this increase was less than that seen in SARD (**Figure 1F**). In contrast, there was only a slight trend to increased Tph17 cells in these non-SARD groups, which was significantly less than that seen in early SARD (**Figure 1F**).

Both Tfh and Tph cells are reported to provide support for differentiation of B cells to Ab-producing plasma cells and/or plasmablasts (17, 32, 33). We previously showed that there is a trend to increased proportions of plasma cells and plasmablasts in ANA^+^ individuals lacking a SARD diagnosis (17), and similar findings were seen in this study (**Supplementary Figure 1**). When all subjects were included, there was a weak correlation between the proportion of Tfh and Tph cells and the proportion of plasma cells and/or plasmablasts. As might be expected based on the literature, the correlation with plasma cells was slightly stronger for Tfh than Tph (Tfh ρ=0.221, p=0.011; Tph ρ=0.210, p=0.016), whereas the opposite was seen for plasmablasts (Tfh ρ=0.164, p=0.059; Tph ρ=0.222, p=0.010).

Age-associated B cells (ABCs) are increased in SLE (34, 35) and have features suggesting that they are precursors of plasmablasts (34, 36). Consistent with previous studies, the levels of these cells were increased in early SLE, and in SARD overall. However, no substantive increases were seen in ANA^+^ individuals lacking a SARD diagnosis. As previously reported, blood ABC levels were significantly correlated with the proportion of plasmablasts, and to a lesser extent, plasma cells (plasmablasts ρ=0.265, p=0.008; plasma cells ρ=0.255, p=0.011) (32). However, in contrast to previous reports, ABC levels correlated with Tfh (ρ=0.270, p=0.007) and not Tph levels.

Taken together, the data indicates that Tfh and Tph cell activation differs between ANA^+^ individuals with and without SARD, with increases in both the Th2 and Th17 subsets of these populations in early SARD patients relative to those lacking a SARD diagnosis.

### T Regulatory Cell Subsets Are Increased in ANA^+^ NS and UCTD, Relative to Early SARD

**Figure 2:**
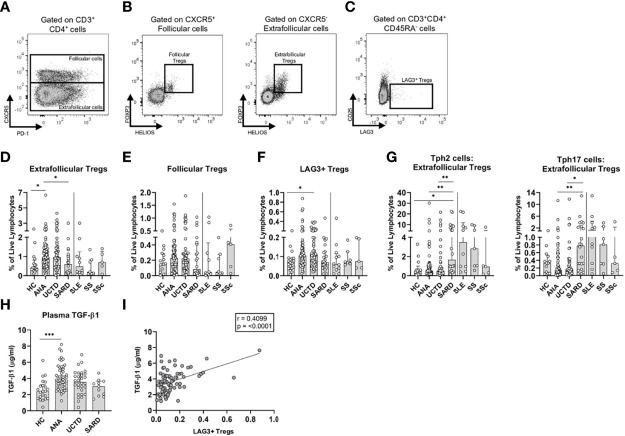
T regulatory (Treg) subsets and transforming growth factor beta-1 (TGF-β1) are increased in anti-nuclear antibody positive (ANA^+^) individuals lacking a systemic autoimmune rheumatic diseases (SARD) diagnosis. **(A)** Gating strategy for identification of (CD3^+^CD4^+^) follicular (CXCR5^+^) and extra-follicular (CXCR5^-^) T cells for a representative ANA^+^ patient. Gating strategy for identification of **(B)** (HELIOS^+^FOXP3^+^) follicular and extra-follicular Tregs and **(C)** memory (CD45RA^-^) LAG3^+^ T regulatory cells (LAG3^+^ Tregs, LAG3^+^CD25^-^). **(D–F)** The proportions of Treg subsets stratified by subject group. **(G)** The ratio of memory T peripheral helper 2 cells to extra-follicular Tregs; and the ratio of memory T peripheral helper 17 cells to extra-follicular Tregs stratified by subject group on a log10 scale. **(H)** Plasma TGF-β1 levels stratified by subject group. **(I)** The correlation between the proportion of memory LAG3^+^ Tregs and TGF-β1 levels. The solid vertical line in each plot separates the groups that were statistically compared to one another from the individual SARD on the right, which were not statistically compared to any group. Bars represent the median with interquartile range. Each data point represents an individual subject. Statistical significance was determined using the Kruskal-Wallis test with Dunn’s *post-hoc* test for multiple comparisons; *p ≤ 0.05, **p ≤ 0.01, ***p ≤ 0.001. The strength of association was determined using a non-parametric Spearman correlation analysis. The solid line of best fit was computed from linear regression. HC, ANA^-^ healthy control; ANA, asymptomatic ANA^+^; UCTD, undifferentiated connective tissue disease; SARD, systemic autoimmune rheumatic disease; SLE, systemic lupus erythematosus; SS, Sjögren’s syndrome; SSc, systemic sclerosis.

Although there is some inconsistency regarding the proportion and function of T regulatory (Treg) cell populations in SARD, possibly due to heterogeneity in defining these populations and the markers used for their identification, available evidence suggests that Treg cells are reduced and/or functionally impaired in SARD patients (37–45). It has also been proposed that Tregs act to prevent symptoms in ANA^+^ individuals lacking a SARD diagnosis (46). To explore whether there are differences in the proportions of various Treg populations between symptomatic and asymptomatic ANA^+^ individuals, we examined extra-follicular, follicular, and LAG3^+^ Treg populations, gated as shown in **Figures 2A–C**. For all three populations, there was a consistent trend to increase in asymptomatic ANA^+^ NS and UCTD patients as compared to ANA^-^ HC and early SARD patients (**Figures 2D–F**), which variably achieved statistical significance. In contrast, these populations were either similar or somewhat reduced in SARD patients as compared to ANA^-^ HC. As a result, there was a significant increase in the ratio of Tph2 and Tph17 cells to extra-follicular Tregs in SARD patients when compared with ANA^+^ individuals lacking a SARD diagnosis (**Figure 2G**).

One of the mechanisms by which Tregs, particularly LAG3^+^ cells, exert their function is through secretion of TGF-β1 (47). Consistent with enhanced immunoregulation in ANA^+^ NS, there were significantly elevated plasma levels of this cytokine relative to ANA^-^ HC (**Figure 2H**), with a progressive trend to normalization in UCTD and SARD patients. As expected, there was a moderate positive correlation between the proportion of LAG3^+^ Tregs, but not extra-follicular or follicular Tregs, and TGF-β1 (**Figure 2I**).

Collectively, these findings suggest that there is a shift from predominant T cell regulation to predominant pro-inflammatory T cell activation that discriminates asymptomatic ANA^+^ NS individuals from early SARD.

### Accumulation of Innate Immune Populations Favoring Production of Pro-Inflammatory Factors Differentiates Early SARD From Asymptomatic ANA^+^ Individuals

**Figure 3:**
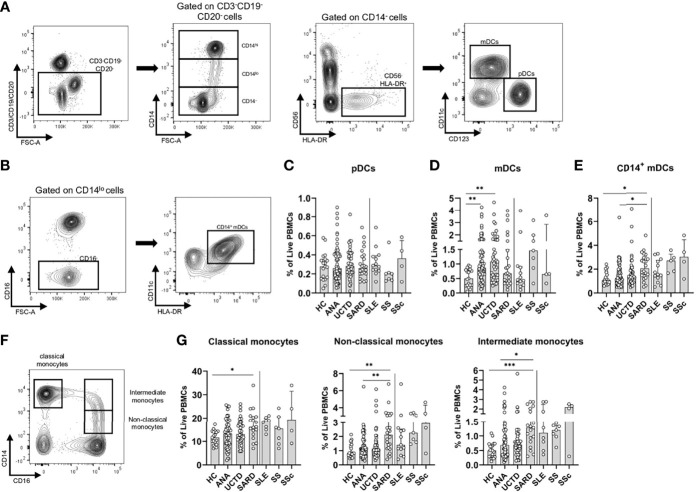
Differences in the frequencies of innate immune populations distinguish anti-nuclear antibody positive (ANA^+^) individuals lacking a systemic autoimmune rheumatic diseases (SARD) diagnosis from early SARD patients. **(A)** Gating strategy for identification of CD14^-^HLA-DR^+^CD56^-^ plasmacytoid dendritic cells (pDCs, CD123^+^CD11c^-^) and myeloid dendritic cells (mDCs, CD123^-^CD11c^+^) from the lineage negative compartment (CD3^-^CD19^-^CD20^-^) in a representative ANA^+^ patient. **(B)** Gating strategy for identification of CD14^+^ mDCs (CD14^lo^CD123^-^CD11c^+^). **(C–E)** The proportion of pDCs, mDCs, and CD14^+^ mDCs stratified by subject group. **(F)** Gating strategy for identification of classical monocytes (CD16^-^CD14^hi^); non-classical monocytes (CD16^+^CD14^lo^); and intermediate monocytes (CD16^+^CD14^-^). **(G)** The proportion of the monocyte subsets stratified by subject group. The solid vertical line in each plot separates the groups that were statistically compared to one another from the individual SARD on the right, which were not statistically compared to any group. Bars represent the median with interquartile range. Each data point represents an individual subject. Statistical significance was determined using the Kruskal-Wallis test with Dunn’s *post-hoc* test for multiple comparisons. *p ≤ 0.05, **p ≤ 0.01, ***p ≤ 0.001. HC, ANA^-^ healthy control; ANA, asymptomatic ANA^+^; UCTD, undifferentiated connective tissue disease; SARD, systemic autoimmune rheumatic disease; SLE, systemic lupus erythematosus; SS, Sjögren’s syndrome; SSc, systemic sclerosis.

Dendritic cells (DC) play an important role in supporting immune activation in SARD, both through the production of type I IFN by plasmacytoid DCs (pDCs) and activation of T cell subsets by myeloid DCs (mDCs). Studies have shown that in SARD patients with active ongoing inflammation, there is a trend to reduced levels of these cells in the peripheral blood, which is associated with their increased localization to the tissues (48–50). To assess how these populations differ between symptomatic and asymptomatic ANA^+^ individuals, pDCs and mDCs were examined (gating shown in **Figures 3A, B**). mDCs were further divided into CD14^+^ and CD14^-^ subsets, as previous studies have shown that CD14^+^ mDCs are expanded in SARD, express a variety of pro-inflammatory cytokines, and are very effective inducers of Th2 and Th17 differentiation (51). As shown in **Figure 3C**, no differences were seen in the proportion of pDCs between any of the ANA^+^ subject sub-groups and ANA^-^ HC. However, there was a significant increase in the proportion of CD14^-^ mDCs in ANA^+^ individuals lacking a SARD diagnosis as compared to ANA^-^ HC, with a trend to decrease in SARD patients as compared to the other ANA^+^ groups (**Figure 3D**). Conversely, the proportion of CD14^+^ mDCs was significantly increased in SARD as compared to both ANA^-^ HC and ANA^+^ NS (**Figure 3E**). These findings suggest that there is a relative depletion of CD14^-^ mDCs and accumulation of the more pro-inflammatory CD14^+^ mDCs in the circulation of patients with early SARD, as compared to ANA^+^ individuals lacking symptoms.

Previous studies indicate that SARD patients have increased proportions of monocytes in their peripheral blood, particularly those of the intermediate and non-classical type (52–55). Non-classical monocytes have been shown to have an increased capacity to secrete pro-inflammatory molecules and present antigens to T cells, as compared to classical monocytes (56, 57). To determine whether similar changes were observed in ANA^+^ individuals lacking a SARD diagnosis, classical (CD14^hi^CD16^-^), non-classical (CD14^lo^CD16^+^) and intermediate monocytes (CD14^hi^CD16^+^), were gated as shown in **Figure 3F**. All three subsets were significantly expanded in early SARD when compared to ANA^-^ HC (**Figure 3G**). Although there was a slight trend to an increase in these populations in ANA^+^ NS and UCTD patients compared to ANA^-^ HC, the proportion of these cells was significantly lower in ANA^+^ NS individuals than in SARD patients (**Figure 3G**). Thus, individuals with SARD show significant expansion of both pro-inflammatory DC and pro-inflammatory monocyte populations that support T cell activation as compared to asymptomatic ANA^+^ individuals.

### Cellular Phenotypes Seen in ANA^+^ Individuals Lacking a SARD Diagnosis Correlate With autoAb and IFN Levels

**Figure 4:**
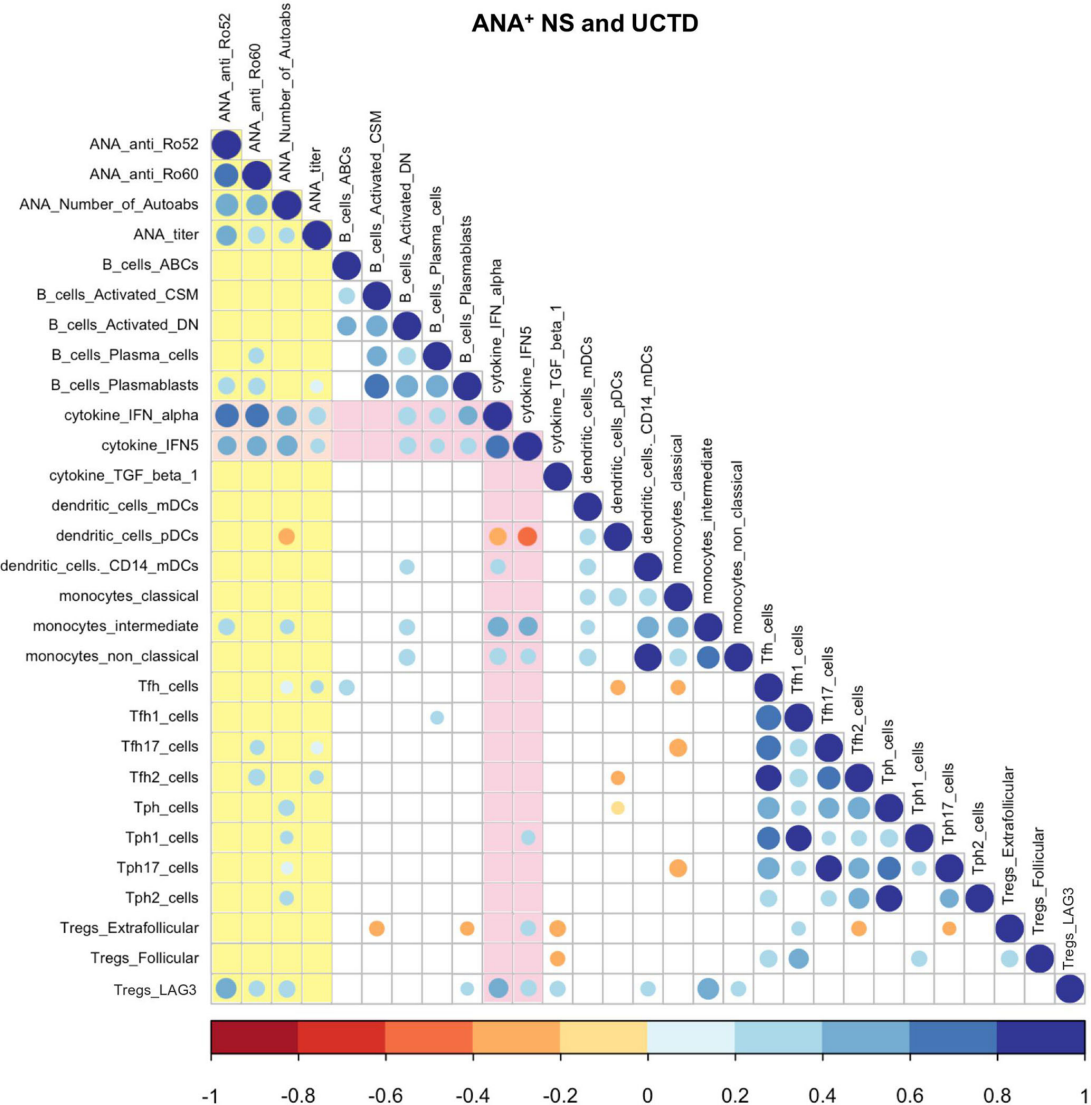
Spearman correlation matrix between cellular and selected serologic/cytokine phenotypes in anti-nuclear antibody positive (ANA^+^) individuals lacking a systemic autoimmune rheumatic diseases (SARD) diagnosis. The color and size of the dots represents the ρ value, with the scales shown at the bottom of each matrix. Non-significant (p ≥ 0.05) correlations are not displayed. Associations with autoAb levels are highlighted in yellow and those with IFN levels are highlighted in pink. ANA, anti-nuclear autoantibody; CSM, class-switched memory; DN, double-negative; IFN, interferon; TGF, transforming growth factor; mDCs, myeloid dendritic cells; pDCs, plasmacytoid dendritic cells; Tfh, T follicular helper; Tph, T peripheral helper; Tregs, T regulatory cells.

As shown in **Supplementary Table 1**, the group of ANA^+^ individuals lacking a SARD diagnosis had significant variation in the type and number of autoAbs seen, as well as the ANA titer. We have previously shown that a subset of these individuals have elevated levels of IFN-induced gene expression in their peripheral blood, as measured by a composite score derived from the levels of 5 IFN-induced genes, termed the IFN5 score (12). We further demonstrated that the levels of this score correlate with the levels of IFN-α, as measured by high sensitivity ELISA (12), as well as anti-Ro60 and -Ro52 antibodies, and that ANA^+^ individuals lacking a SARD diagnosis with high levels of anti-Ro52 antibodies or IFN-α are at an increased risk of clinical progression over the subsequent 2 years (22, 58). To investigate the association between these serologic changes and the peripheral blood cellular profile in these individuals, a Spearman correlation matrix was produced (**Figure 4**). Although **Figure 4** shows the data for the pooled analysis of all ANA^+^ individuals lacking a SARD diagnosis, very similar results were observed when ANA^+^ NS and UCTD patients were examined independently (**Supplemental Material**; **Figure 2**).

As noted in our previous study, there was a moderate positive correlation between two markers of IFN levels, the IFN5 score and/or serum levels of IFN-α, and all of the serologic markers of autoAb production (17). IFN levels also correlated, moderately to strongly, with multiple markers of B cell activation, including activated memory B cell subsets and plasmablasts/plasma cells. This finding is compatible with previous work indicating that IFN acts to enhance B cell activation and differentiation to Ab-producing cells (59–62), and suggests that it may play an important role in driving autoAb production in ANA^+^ individuals lacking a SARD diagnosis. The observation that the levels of plasmablasts/plasma cells correlate with serologic markers of autoAb production supports this concept. AutoAb production also demonstrated a weak correlation with Tfh and Tph cells, together with several of the subsets within these populations, consistent with the role of these cells in supporting Ab production. In general, the proportions of these T cells and their subpopulations did not correlate with IFN levels.

Unlike the pro-inflammatory T cell subsets, the proportion of LAG3^+^ Tregs positively correlated with both autoAb and IFN levels, suggesting that the same immune processes that lead to activation of other immune populations may act to expand LAG3^+^ Tregs, which may act in turn to suppress development of symptomatic autoimmunity. In contrast, the proportions of extra-follicular and follicular Tregs did not correlate with autoAb production, and in the case of extra-follicular Tregs demonstrated negative correlations with some of the activated immune populations.

Although the majority of innate immune subsets did not correlate with autoAb production, a number of populations correlated with IFN levels. Notably, the proportion of pDCs correlated inversely with markers of elevated IFN levels, suggesting that, similar to what is observed in SARD (48–50), pDCs are depleted from the circulation when high levels of IFN-α are produced, possibly as a result of recruitment to the tissues. In contrast, the levels of CD14^+^ mDCs, intermediate monocytes, and non-classical monocytes all showed a moderate positive correlation with IFN levels. These findings suggest that one of the mechanisms by which high levels of IFN may promote progression is through facilitating development of these pro-inflammatory innate immune populations.

### Progressors Have More B and T Cell Activation Than Non-Progressors

As some of the immune cell populations correlated with elevated autoAb/IFN levels, which had been reported to be associated with an increased risk of clinical progression (22, 58, 63), it was of interest to us to determine the cellular immunologic features that distinguish ANA^+^ individuals without SARD who will progress clinically from those who will not. To address this question, yearly longitudinal follow-up was offered to all of these individuals, with the option of attending clinic earlier if new symptoms developed. At present, there are 20 ANA^+^ individuals who demonstrated symptomatic progression within 2 years of recruitment, defined as the development of new SARD diagnostic criteria or new organ involvement characteristic for SARD. Non-progressors were defined as participants who were followed for at least two years and remained stable without development of new symptoms during that period. The clinical characteristics of the progressors and non-progressors are outlined in **Supplementary Table 2** and an outline of disease progression in patients who progressed is given in **Supplementary Table 3**.

**Figure 5:**
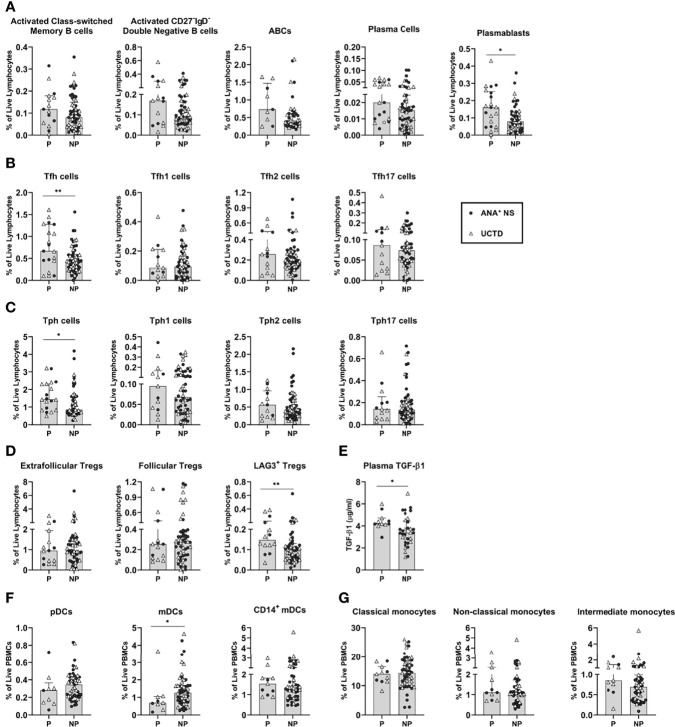
Antinuclear antibody positive (ANA^+^) individuals lacking a systemic autoimmune rheumatic diseases (SARD) diagnosis who demonstrated symptomatic progression demonstrate differences in adaptive and innate immune populations, relative to non-progressors. All graphs compare progressors and non-progressors at baseline (initial assessment). Patients diagnosed as ANA^+^ NS or UCTD at initial assessment are represented by the closed circles and the open triangles, respectively. **(A)** B cell subsets. **(B, C)** T helper cell subsets. **(D)** T regulatory cell subsets. **(E)** Plasma transforming growth factor beta-1 (TGF-β1) levels. **(F)** Dendritic cell subsets. **(G)** Monocyte subsets. Bars represent the median with interquartile range. Each data point represents an individual subject. For each set of comparisons, statistical significance was determined using the Mann-Whitney test. *p ≤ 0.05, **p ≤ 0.01. P, Progressors; NP, Non-Progressors.

As shown in **Figure 5A**, within the B cell lineage, progressors had a significant increase in the proportion of plasmablasts as compared to non-progressors. Trends to increased proportions of activated class-switched memory and CD27^-^IgD^-^ double negative memory B cells, as well as ABCs and plasma cells, were also seen in progressors. These findings suggest that higher levels of B cell activation may be associated with an increased likelihood of progression.

Similar findings were observed for T cells, with higher percentages of Tfh and Tph cells in progressors as compared to non-progressors (**Figures 5B, C**). This increase was not associated with an expansion of any particular cytokine-producing subset. Although there were trends to an increase in the Tfh2, Tfh17, Tph1 and Tph2 subsets in progressors as compared to non-progressors, none of these achieved statistical significance. Thus, despite evidence for higher levels of Th2- and Th17-type cells in early SARD, increased levels of these populations do not appear to occur prior to or predict symptomatic progression.

Although the levels of the various Treg subsets were generally reduced in SARD as compared to ANA^+^ individuals lacking a SARD diagnosis, no differences were seen in the proportions of extra-follicular or follicular Tregs between progressors and non-progressors (**Figure 5D**). However, there were significantly higher levels of LAG3^+^ Tregs and TGF-ß1 in progressors when compared with non-progressors (**Figures 5D, E**). These findings suggest that the induced T regulatory pathway appears to be activated and expanded in progressors, but ultimately fails to prevent development of symptomatic autoimmunity.

In contrast to the findings observed for adaptive immune populations, the majority of innate immune populations showed no differences between progressors and non-progressors. A significant difference was only observed for the CD14^-^ mDC population, which was reduced in progressors relative to non-progressors, mirroring the difference observed between SARD and ANA^+^ individuals lacking a SARD diagnosis (**Figure 5F**). Very minor trends to decreased pDCs and to increased CD14^+^ mDCs and intermediate monocytes were also seen in progressors (**Figures 5F, G**). Thus, significant accumulation of pro-inflammatory monocytes/DC populations does not appear to precede clinical progression.

## Discussion

While a considerable number of studies have examined the cellular immunologic changes in patients with well-established SARD, often on treatment, studies examining these immunologic changes in ANA^+^ individuals lacking a SARD diagnosis are scarce. In a previous study examining predominantly T and B cell subsets, we found that many of the changes ascribed to SARD are also seen in asymptomatic ANA^+^ individuals (ie. lacking SARD symptoms), suggesting that they are associated with the development of benign autoimmunity rather than the transition to symptomatic disease (17). These findings were validated in the current study, in a largely independent cohort, indicating the robustness of this phenotype. However, it remained to be determined what the key differences were between symptomatic and asymptomatic ANA^+^ individuals. Here we show, by performing a more in-depth analysis of T helper and regulatory cells together with innate immune populations, that these key differences lie in the balance between pro-inflammatory and regulatory immune cell subsets.

We have previously shown that Tfh cells are increased in ANA^+^ NS individuals (17). We report here that this increase is predominantly due to an increase in Th2 cells and that there is a similar increase in Tph2 cells. These findings indicate that both germinal center and extra-follicular T cell responses are enhanced in ANA^+^ NS, and given their correlation with autoAb levels, support autoAb production. Currently, the tissues where the extra-follicular T cell response arise are unknown. The observation that Th2 cells are increased in asymptomatic ANA^+^ individuals, most of whom will never develop SARD, is consistent with previous work showing small but significantly elevated levels of Th1- and Th2-associated cytokines in these individuals (46) and studies showing that these cytokines can be seen years in advance of the transition to disease in SLE patients (64–66). However, in contrast to these serum cytokine studies, increases in circulating Th1 cells were not seen in the current study, nor in our previous study where we examined IFN-γ-producing cells in the CD4^+^ T cell compartment (17). The reason for this disparity is unclear; however, it is possible that cytokine-producing Th1 cells are activated in ANA^+^ NS individuals but remain localized within the tissues, and thus may only be detectable in the circulation through their cytokine secretion.

SARD patients had increased levels of Tph cells and a trend to increased Tfh cells, with increases in both the Th2- and Th17-subsets of these populations, relative to ANA^+^ NS and UCTD patients. These findings suggest that the transition to SARD is associated with increases in the T cell populations that support B cell differentiation to Ab-producing cells. This observation is compatible with previous studies by ourselves and others showing that the number of anti-nuclear autoAbs and/or titers of autoAbs are higher in early SARD than in ANA^+^ individuals lacking a SARD diagnosis (22, 67, 68). In SLE, it has previously been shown that the transition to disease is associated with progressive increases in T cell-derived cytokines, with IL-17 in particular increasing concurrent with disease onset (64). Our findings provide additional support for the concept that significant increases in the Th17-type cells occur concomitantly with early disease, and indicates that this feature extends to the other SARD conditions.

T regulatory cell populations were highest in ANA^+^ NS and appeared to drop to more normal levels in SARD, suggesting that these cells may be actively regulating inflammation to prevent symptomatic disease in ANA^+^ NS. Previous studies examining the cytokine profile of asymptomatic ANA^+^ individuals or SLE patients prior to their transition to symptomatic disease reached a similar conclusion (46, 64). As was seen in those studies, we found that the levels of TGF-ß1 were increased in ANA^+^ NS patients as compared to ANA^-^ healthy controls, and normalized in SARD patients. However, the Treg populations that accompanied these increases were not examined in the earlier studies. Here, we show that ANA^+^ NS and UCTD patients have increases in multiple Treg populations, but only the LAG3^+^ population correlates with TGF-ß1. This observation is compatible with the function of LAG3^+^ Tregs, which have been shown to regulate autoimmunity through secretion of IL-10 and TGF-ß1, as well as through direct cellular contact (47). Notably, LAG3^+^ Tregs are induced in response to multiple environmental stimuli at barrier sites such as the gut, respiratory tract and skin, and have been shown to migrate to remote sites of autoimmune inflammation (69). Whether the expansion of this population indicates a role for environmental triggers in the development of autoimmunity in ANA^+^ NS is currently unknown.

The shift in the balance of Treg to Tfh/Tph cells in early SARD, as compared to ANA^+^ individuals lacking a SARD diagnosis, indicates that the onset of symptomatic autoimmunity is accompanied by a shift from predominant immunoregulation to a more pro-inflammatory pattern. A similar type of shift has been reported for UCTD patients as they transition to SARD, with an increase in the ratio of Th17 to Treg cells (70). The immune mechanisms leading to this shift remain to be definitively determined; however, one possibility is that the expansion of CD14^+^ mDCs seen in SARD facilitates this shift. In SLE, this population has been shown to have an enhanced ability to support Th17 differentiation and, through OX40L expression, to augment Tfh cell differentiation and impair Treg function (51, 71). The non-classical and intermediate monocytes that are expanded in SARD have also been reported to support T cell activation/differentiation (56, 57). Alternatively, the balance of Treg to Tfh/Tph cells could be affected by changes in immune function at barrier sites, such as the gastrointestinal tract. Previous studies have shown that there are alterations in the gut microbiome in SARD that can be associated with enhanced gut permeability (72), which have been shown to facilitate a shift in the Treg to Th17 balance (73, 74).

In ANA^+^ individuals lacking a SARD diagnosis, there was an inverse correlation between the levels of pDCs and serum levels of IFN-α and IFN-induced gene expression. These findings contrast with the results of a previous study of ANA^+^ ‘at-risk’ individuals where decreased levels of pDCs were seen when compared with healthy controls (75). In that study, there was no correlation between the levels of pDCs and peripheral blood IFN-induced gene expression. Based upon this lack of correlation, together with RNAseq and functional data suggesting that the pDCs are functionally impaired in ‘at risk’ individuals, it was argued that pDCs are not a source of the IFN that induces the altered gene expression in the peripheral blood. Our findings argue for an alternate explanation for this lack of responsiveness, specifically that it reflects prior activation of this population. Along these lines, we and others have previously shown that pDCs transiently produce IFN-α and then become refractory to further activation with Toll-like receptor (TLR) stimulation (76, 77), a phenomenon termed TLR tolerance. TLR signaling in pDCs also induces their migration to the tissues, which may account for their depletion from the blood.

Comparison of progressors and non-progressors prior to progression indicated that progressors had elevated levels of B and T cell activation, with changes reflecting increased follicular and extra-follicular (tissue) responses, as compared to non-progressors. Progressors also had increases in the proportion of LAG3^+^ Treg cells and TGF-ß1, suggesting that these cells are expanded during the immune response that leads to progression, but fail to prevent development of symptoms. Whether this failure results from impaired function of this or other Treg populations, as has been reported for SARD (40, 43–45, 71, 78), remains to be determined. Surprisingly, progressors had reduced levels of mDCs as compared to non-progressors. mDCs shuttle from the blood stream through the tissues and are retained in the tissue and/or draining lymph nodes when there is localized inflammation. Thus, the depletion of these cells may indicate the presence of sub-clinical inflammation prior to the onset of overt clinical symptoms in progressors.

In summary, we have identified a number of immunologic features that discriminate asymptomatic ANA^+^ individuals from early SARD patients, and ANA^+^ symptom progressors from non-progressors. Our findings provide insight into the immune mechanisms that lead to clinical symptoms in SARD, and raise the possibility of targeting these mechanisms to block development of SARD.

## Data Availability Statement

The raw data supporting the conclusions of this article will be made available by the authors, without undue reservation.

## Ethics Statement

The studies involving human participants were reviewed and approved by Research Ethics Boards of the University Health Network and Mount Sinai Hospital. The patients/participants provided their written informed consent to participate in this study.

## Author Contributions

All authors were involved in drafting the article or revising it critically for important intellectual content, and all authors approved the final version to be published. JW and RG had full access to all of the data in the study and take responsibility for the integrity of the data and the accuracy of the data analysis. Study conception and design: RG, EV, KM, and JW. Acquisition of data: RG, EV, KM, DB, MK, CM-G, CN, SJ, LH, ZA, ZT, DB, AB, and JW. Analysis and interpretation of data: RG, EV, CM-G, CN, and JW. All authors contributed to the article and approved the submitted version.

## Funding

The study was funded by a grant from the Canadian Institutes of Health Research (CIHR, FRN 159563) to JW. JW receives salary support from a Pfizer Chair Research Award, the Arthritis Centre of Excellence, and the Schroeder Arthritis Institute. The funder, Pfizer Chair Research Award, was not involved in the study design, collection, analysis, interpretation of data, the writing of this article or the decision to submit it for publication. SJ is supported by a CIHR salary support award. LH is the recipient of a The Arthritis Society Stars Career Development Award. RG was supported by a Canada Graduate Scholarship (CGS) CIHR Award and Queen Elizabeth II Graduate Scholarship in Science and Technology (QEII-GSST). EV received support from an Ontario Graduate Scholarship Award. CN is the recipient of a CGS-CIHR award. CM-G is supported by a PhD salary award from The Arthritis Society. The funders were not involved in the study design, collection, analysis, interpretation of data, the writing of this article or the decision to submit it for publication.

## Conflict of Interest

The authors declare that the research was conducted in the absence of any commercial or financial relationships that could be construed as a potential conflict of interest.

## Publisher’s Note

All claims expressed in this article are solely those of the authors and do not necessarily represent those of their affiliated organizations, or those of the publisher, the editors and the reviewers. Any product that may be evaluated in this article, or claim that may be made by its manufacturer, is not guaranteed or endorsed by the publisher.

## Abbreviations

ANA, Anti-nuclear antibody; Ab, Antibody; ELISA, Enzyme-linked immunosorbent assay; HC, Healthy control; IFN, Interferon; IFN-α, Interferon-alpha; IL, Interleukin; mDC, myeloid dendritic cell; NS; asymptomatic; pDC, plasmacytoid dendritic cell; PBMC, Peripheral blood mononuclear cell; SARD, Systemic autoimmune rheumatic disease; SjD, Sjogren’s Disease; SLE, Systemic lupus erythematosus; SSc, Systemic sclerosis; Tfh, T follicular helper;TGF-ß1, Transforming growth factor beta-1; Tph; T peripheral helper; Treg, T regulatory; UCTD, Undifferentiated connective tissue disease.
