# Supplementary material for: Altered Balance of Pro-Inflammatory Immune Cells to T Regulatory Cells Differentiates Symptomatic From Asymptomatic Individuals With Anti-Nuclear Antibodies
Source: Front Immunol. 2022 Jun 30;13:886442. doi: 10.3389/fimmu.2022.886442 (PMC9279569; doi:10.3389/fimmu.2022.886442)
Supplement: Supplementary file 1 [file DataSheet_1.docx]

Supplementary Figures and Tables

**Supplementary Table 1:** Study Participant Characteristics

*Abbreviations: ANA*, anti-nuclear antibody; *HC*, healthy control; *NS,* asymptomatic; *UCTD*, undifferentiated connective tissue disease; *SARD*, systemic autoimmune rheumatic disease; *SLE,* systemic lupus erythematosus; *SS*, Sjögren’s syndrome; *SSc,* systemic sclerosis; *N,* number; *SD*, standard deviation; *Abs*, antibodies; *Sm*, Smith; *RNP*, ribonuclear protein

*The age of one healthy control is unknown

^a^Values significantly (p < 0.05) different from ANA^−^ HC are in bold

^b^Values significantly (p < 0.05) different from SARD

|  | ANA^-^ HC  (*n* = 22) | ANA^+^ NS  (*n* = 72) | UCTD  (*n* = 52) | SARD  (*n* = 27) | SLE  (*n* = 15) | SS  (*n* = 7) | SSc  (*n* = 5) |
| --- | --- | --- | --- | --- | --- | --- | --- |
| Female sex, *n* (%) | 21 (95.5) | 68 (94.4) | 47 (90.4) | 24 (89) | 15 (100) | 5 (71.4) | 4 (80) |
| Age, years, mean ± SD | 32.14 ± 9.75^*^ | **42.07 ± 14.17^a^** | **43.77 ± 12.93** | 40.89 ± 17.73 | 32.87 ± 13.53 | 52.86 ± 20.60 | 48.2 ± 14.41 |
| Ethnicity, n (%)  Caucasian  Afro-Caribbean  East Asian  South Asian  Hispanic  Other | 12 (54.5)  0 (0)  4 (18.2)  4 (18.2)  2 (9.1)  0 | 38 (52.8)  5 (6.9)  13 (18)  3 (4.2)  3 (4.2)  10 (13.9) | 38 (73.1)  2 (3.8)  4 (7.7)  3 (5.8)  3 (5.8)  2 (3.8) | 16 (59.3)  1 (3.7)  4 (14.8)  1 (3.7)  3 (11.1)  2 (7.4) | 7 (46.7)  1 (6.7)  3 (20)  0 (0)  2 (13.3)  2 (13.3) | 5 (71.4)  0 (0)  1 (14.3)  1 (14.3)  0 (0)  0 (0) | 4 (80)  0 (0)  0 (0)  0 (0)  1 (20)  0 (0) |
| Anti-malarials, *n* (%) | N/A | 1 (1.4)^b^ | 13 (25) | 8 (29.6) | 7 (46.7) | 1 (14.3) | 0 (0) |
| ANA titer, n (%)  1:80  1:160  1:320  1:640  >1:160  ANA titer, median | 0 (0)  0 (0)  0 (0)  0 (0)  0 (0)  0 | 5 (6.9)  16 (22.2)  12 (16.7)  24 (33.3)  15 (20.8)  1:640 | 2 (3.9)  7 (13.5)  13 (25)  15 (28.8)  15 (28.8)  1:640 | 3 (11.1)  1 (3.7)  2 (7.4)  7 (25.9)  14 (51.9)  > 1:640 | 2 (13.3)  1 (6.7)  1 (6.7)  6 (40)  5 (33.3)  1:640 | 1 (14.3)  0 (0)  0 (0)  0 (0)  6 (85.7)  > 1:640 | 0 (0)  0 (0)  1 (20)  1 (20)  3 (60)  > 1:640 |
| Specific AutoAbs, n (%)  dsDNA  Ro  La  Sm  Sm/RNP  RNP  Scl-70  Jo-1  Centromere  Chromatin  Ribosomal P  Specific AutoAbs mean ± SD | 0 (0)  0 (0)  0 (0)  0 (0)  0 (0)  0 (0)  0 (0)  0 (0)  0 (0)  0 (0)  0 (0)  N/A | 8 (11.1)  27 (37.5)  6 (8.3)  1 (1.4)^b^  2 (2.8)^b^  6 (8.3)  4 (5.6)  0 (0)  0 (0)  1 (1.4)^b^  1 (1.4)  0.78 ± 0.94^b^ | 0 (0)  19 (36.5)  4 (7.7)  2 (3.8)^b^  6 (11.5)  8 (15.4)  4 (7.7)  0 (0)  8 (15.4)  6 (11.5)  0 (0)  1.10 ± 1.12^b^ | 4 (14.8)  14 (51.9)  6 (22.2)  5 (18.5)  7 (25.9)  7 (25.9)  0 (0)  1 (3.7)  2 (7.4)  7 (25.9)  0 (0)  1.96 ± 1.43 | 4 (26.7)  5 (43.3)  1 (6.7)  5 (33.3)  7 (46.7)  6 (40)  0 (0)  1 (6.7)  0 (0)  6 (40)  0 (0)  2.33 ± 1.68 | 0 (0)  7 (100)  5 (71.4)  0 (0)  0 (0)  0 (0)  0 (0)  0 (0)  0 (0)  1 (14.3)  0 (0)  1.86 ± 0.69 | 0 (0)  2 (40)  0 (0)  0 (0)  0 (0)  1 (20)  0 (0)  0 (0)  2 (40)  0 (0)  0 (0)  1.0 ± 1.00 |

**Supplementary Table 2:** Progressors and Non-Progressors Characteristics at Baseline

|  | Progressors  (*n* = 20) | Non-Progressors  (*n* = 60) |
| --- | --- | --- |
| Female, sex, *n* (%) | 17 (85) | 55 (92) |
| Age, years, mean ± SD | 47.30 ± 11.25 | 43.53 ± 14.21 |
| Ethnicity, Caucasian, *n* (%) | 12 (60) | 41 (68) |
| Anti-malarials, *n* (%) | 3 (15) | 11 (18.3) |
| ANA titer, median | 4 | 4 |
| Specific AutoAbs, mean ± SD | **1.60 ± 1.57^a^** | 0.77 ± 0.79 |
| Ro52 titer, mean ± SD | **12.86 ± 3.48^a^** | 8.61 ± 4.09 |
| IFN5 score, mean ± SD | 55.60 ± 8.93 | 53.54 ± 7.00 |
| IFN-α (pg/ml), mean ± SD | 0.89 ± 1.88 | 1.43 ± 6.20 |

*Abbreviations:* *n,* number; *SD*, standard deviation; *ANA*, anti-nuclear antibody; *Abs*, antibodies; *IFN5*, Interferon 5*; IFN-α*, Interferon alpha

^a^Values significantly (p < 0.05) different from Non-Progressors are in bold

^b^The Ro52 titre is unknown for 8 Progressors and 23 Non-Progressors

^c^The IFN5 score is unknown for 3 Progressors and 13 Non-Progressors

^d^The IFN-α level is unknown for 4 Progressors and 14 Non-Progressors

**Supplementary Table 3:** Outline of Disease Progression in Progressors

| **ANA^+^NS (9)** | | | |
| --- | --- | --- | --- |
| **Baseline clinical criteria** | **Sex** | **New clinical criteria** | **Final diagnosis** |
| Nil | Female | Arthritis, inflammatory rash,  Raynaud's phenomenon | SLE |
| Nil | Female | Alopecia, photosensitivity,  Raynaud's phenomenon | SLE |
| Nil | Female | Arthritis, Raynaud’s phenomenon | UCTD |
| Nil | Female | Unilateral Schirmer ≤ 5 mm/5min, Ocular and Oral symptoms | SS |
| Nil | Female | Bilateral Schirmer ≤ 5 mm/5min, Ocular and Oral symptoms | SS |
| Nil | Male | Thrombocytopenia | UCTD |
| Nil | Female | Unilateral Schirmer ≤ 5 mm/5min | UCTD |
| Nil | Female | Arthritis | UCTD |
| Nil | Female | Arthritis, increased dsDNA, nephritis | SLE |
| **UCTD (11)** | | | |
| **Baseline criteria** | **Sex** | **New criteria** | **Final diagnosis** |
| Raynaud's phenomenon, abnormal nailfold capillaries | Male | Puffy fingers | UCTD |
| Raynaud's phenomenon | Female | Nasal ulcers, arthritis | SLE |
| Raynaud's phenomenon,  esophageal dysmotility | Female | Lower intestinal tract dysmotility | UCTD |
| Abnormal salivary pool, peripheral neuropathy | Female | Arthritis | UCTD |
| Raynaud's phenomenon, abnormal nailfold capillaries, puffy fingers | Female | Fingertip pitting | SSc |
| Photosensitivity | Female | Parotitis | UCTD |
| Raynaud's phenomenon | Male | Puffy fingers | UCTD |
| Bilateral Schirmer ≤ 5 mm/5min, reduced salivary flow | Female | Arthritis | UCTD |
| Raynaud’s phenomen, abnormal nailfold capillaries | Female | Puffy Digits | SSc |
| Unilateral Schirmer ≤ 5 mm/5min, Ocular symptoms | Female | Oral Symptoms | SS |

*Abbreviations:* *ANA^+^ NS*, anti-nuclear antibody positive asymptomatic; *SLE*, systemic lupus erythematosus; *UCTD*, undifferentiated connective tissue disease; *SS*, Sjögren’s syndrome; *SSc*, systemic sclerosis


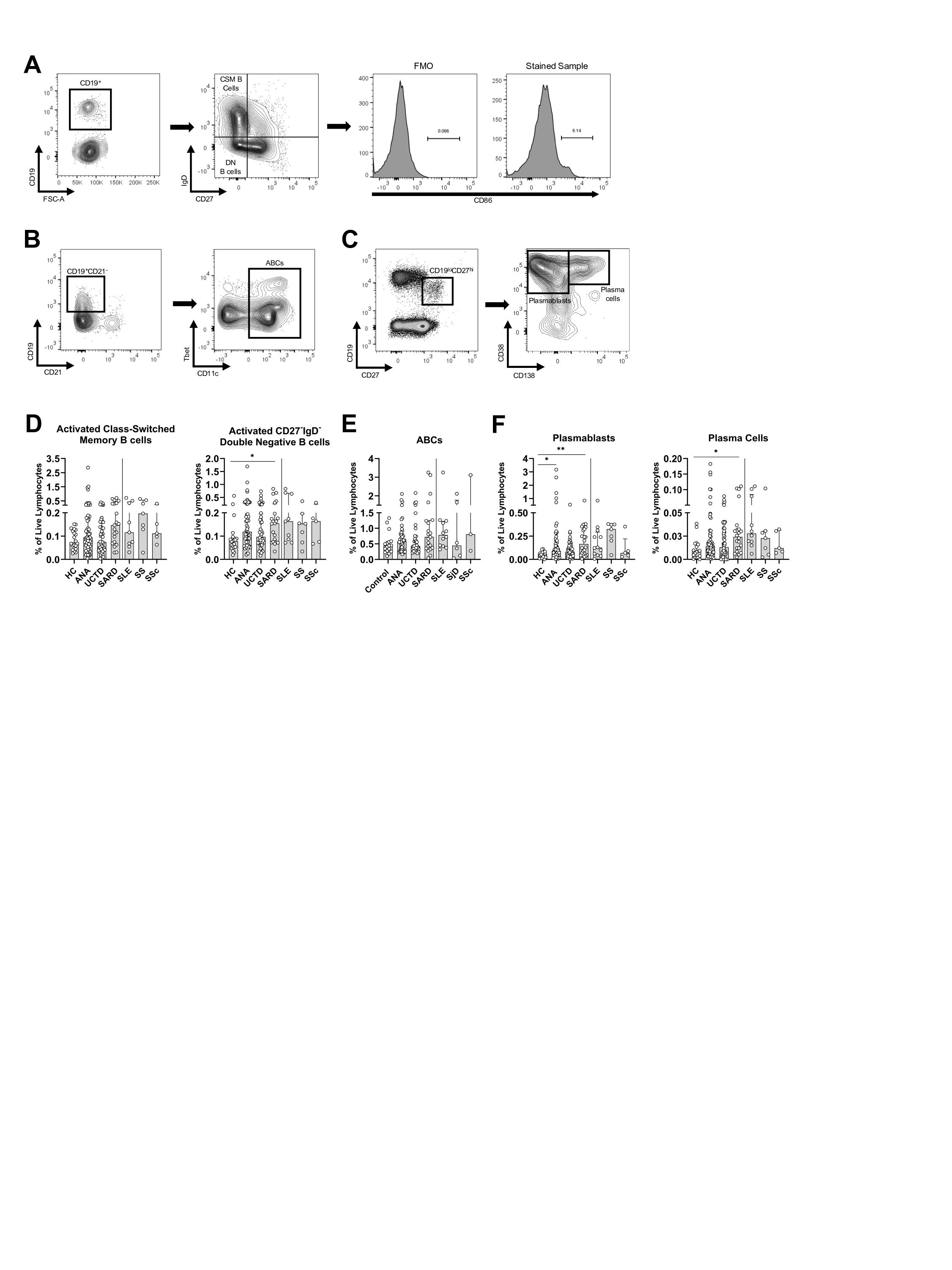


**Supplementary Figure 1. B cell subsets associated with autoimmunity and involved in auto-antibody production are significantly expanded in systemic autoimmune rheumatic diseases (SARD) patients**. **(A)** Gating strategy for identification of activated (CD86^+^) class-switched memory (CSM) B cells (CD27^-^IgD^+^) and activated double negative B cells (CD27^-^IgD^-^) from the B cell compartment (CD19^+^) in a representative anti-nuclear antibody positive (ANA^+^) patient. **(B)** Gating strategy for identification of age-associated B cells (ABCs, CD11c^+^) from the activated B cells compartment (CD21^-^CD19^+^). **(C)** Gating strategy for identification of plasmablasts (PBs, CD138^-^CD38^+^) and plasma cells (PCs, CD138^+^CD38^+^) from the memory B cell compartment (CD27^hi^CD19^lo^). **(D-F)** The proportion of the B cell subsets stratified by subject group. The solid vertical line in each plot separates the left side groups, which were statistically compared to one another, from the individual SARD on the right, which were not statistically compared to any group. Bars represent the median with interquartile range. Each data point represents an individual patient or HC. For each set of comparisons statistical significance was determined using the Kruskal-Wallis test with Dunn’s post-hoc test for multiple comparisons. *p ≤ 0.05, **p ≤ 0.01. HC, ANA^-^ healthy control; ANA, asymptomatic ANA^+^; UCTD, undifferentiated connective tissue disease; SARD, systemic autoimmune rheumatic disease; SLE, systemic lupus erythematosus; SS, Sjögren’s syndrome; SSc, systemic sclerosis


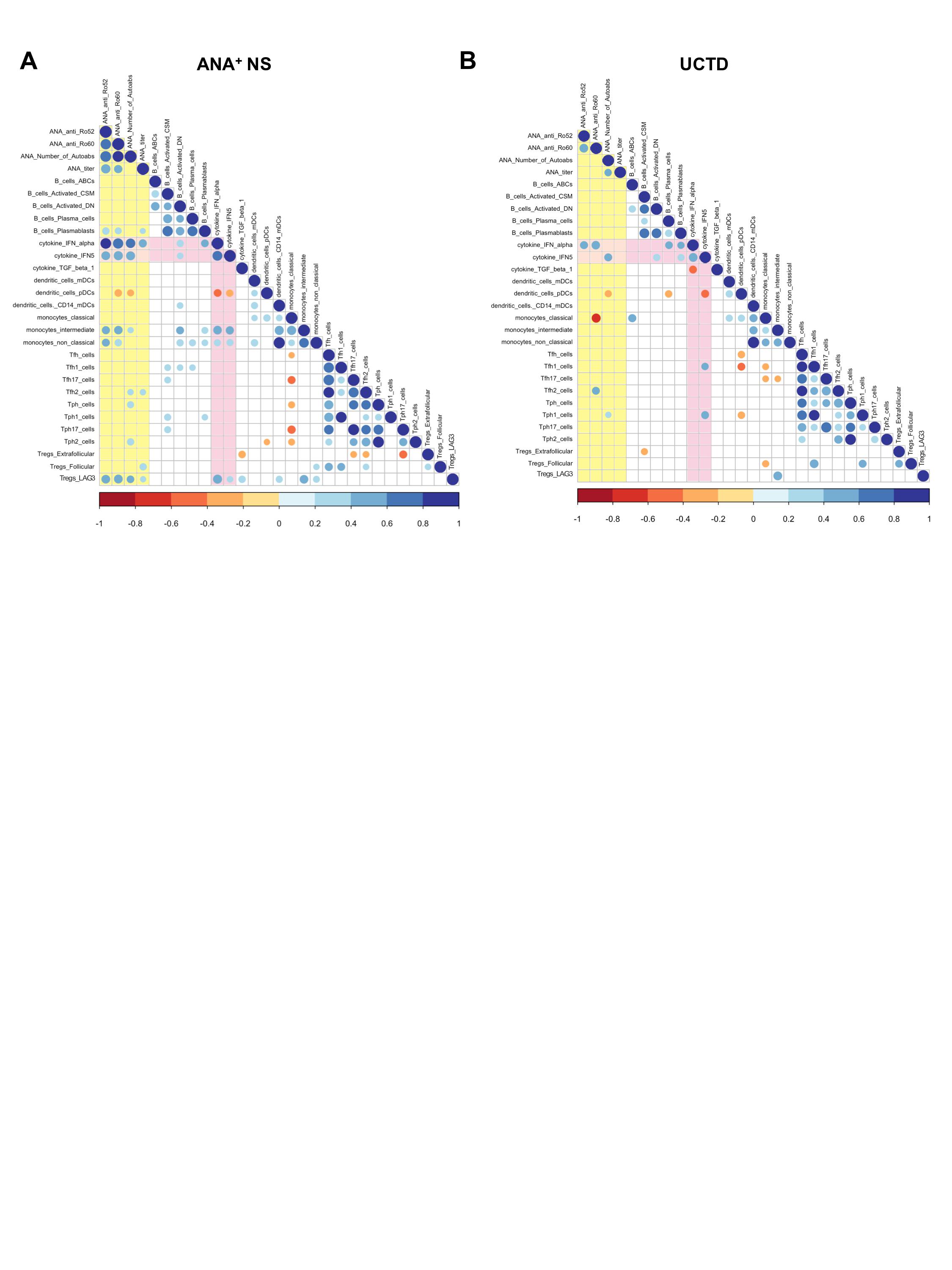


**Supplementary Figure 2. Spearman correlation matrix between cellular and selected serologic/cytokine phenotypes in anti-nuclear antibody positive (ANA^+^) individuals lacking a systemic autoimmune rheumatic diseases (SARD) diagnosis. (A)** Spearman correlation matrix for asymptomatic ANA^+^ (ANA^+^ NS) individuals **(B)** Spearman correlation matrix for undifferentiated connective tissue disease (UCTD) patients. The color and size of the dots represents the ρ value, with the scales shown at the bottom of each matrix. Non-significant (p ≥ 0.05) correlations are not displayed. ANA, anti-nuclear autoantibody; CSM, class-switched memory; DN, double-negative; IFN, interferon; TGF, transforming growth factor; mDCs, myeloid dendritic cells; pDCs, plasmacytoid dendritic cells, Tfh, T follicular helper; Tph, T peripheral helper; Tregs, T regulatory cells
